# MicroRNA-191-5p diminished sepsis-induced acute kidney injury through targeting oxidative stress responsive 1 in rat models

**DOI:** 10.1042/BSR20190548

**Published:** 2019-08-13

**Authors:** Yi Qin, Guizhen Wang, Zhiyong Peng

**Affiliations:** 1 Department of Critical Care Medicine, Zhongnan Hospital of Wuhan University, No. 169 Donghu Road, Wuhan 430071, Hubei Province, China; 2 Department of Emergency, Tenth People’s Hospital of Tongji University, No. 301 Yanchang Road, Shanghai, 200072, China

**Keywords:** Apoptosis, Cecal ligation and puncture, Inflammatory cytokines, Septic acute kidney injury

## Abstract

There is no effective treatment for septic acute kidney injury (AKI), which is considered a major public health concern in today’s world. Here, we studied the functions of miR-191-5p in septic AKI. MiR-191-5p mimic or mimic control was injected into rats from caudal vein before cecal ligation and puncture (CLP) surgery. Part of kidney tissues was stained by Hematoxylin and Eosin (H&E) for histological examination. The levels of serum cytokines were evaluated using enzyme-linked immunosorbent assay (ELISA). For cell transfection, renal cells were isolated from the kidneys of CLP rat model injected with mimic control and miR-191-5p mimic. With TargetScan prediction, serine/threonine-protein kinase OSR1 was identified as a target of miR-191-5p. Oxidative stress responsive 1 (OXSR1) overexpression vector was transfected into renal cells. Cell viability and apoptosis rate were determined by Cell Counting Kit-8 (CCK-8) and flow cytometry, respectively. We additionally measured the phosphorylation levels of p38 and p65. We found that the injection of miR-191-5p mimic could observably inhibit renal injury scores, and inhibit inflammatory cytokine productions and apoptotic protein levels in septic rats. After being transfected with OXSR1, the apoptosis rates and expressions of B-cell lymphoma-2 (Bcl-2), down-regulated Bax and Cleaved caspase-3 (C caspase-3) indicated overexpressed OXSR1 contributed to cell apoptosis. The up-regulated protein levels of p-p38 and p-p65 may suggest the involvement of p38 MAPK/NF-κB signaling pathway in the functions of OXSR1. Our results showed that the protective effects of miR-191-5p on kidney tissues of septic rats may rely on the repression of OXSR1.

## Introduction

Sepsis is a condition characterized by a systemic inflammatory response syndrome (SIRS) triggered by infections of bacteria, virus or fungus [1,2]. The host response to infections causes the failure of multiple organs, the kidney is one of the most commonly affected organs [3]. Septic acute kidney injury (AKI) is one of the most severe and frequent complications of sepsis [4]. More than 50% of patients with sepsis develop AKI, and sepsis patients with AKI had a higher mortality rate than patients with sepsis-non AKI [5,6]. Recent studies indicated that the pathogenesis of sepsis-induced AKI contains a series of complex interactions between the dysfunction of vascular endothelial cells, inflammatory response and tubular cell apoptosis [7–9], however, efforts to translate the findings from bench to bedside in clinical trials have been proven to be failed. Therefore, a more complete understanding of the pathogenesis of septic kidney injury is required for developing more effective therapeutic strategies.

MicroRNAs (miRNAs) are a class of small and noncoding RNA molecules which function as negative regulators of post-transcriptional expression of target genes through targeting their mRNAs [10]. Recently, the number of studies about the functions of miRNAs in AKI progression is increasing [11], but most of the work published is focused on ischemic AKI, the data available about miRNA regulation in septic AKI are very limited [12]. In 2017, Fu et al. [13] demonstrated that miR-21 could obviously improve the kidney injury indexes in sepsis-induced AKI rat models through protecting kidney cells from sepsis-induced apoptosis. Furthermore, miR-10-5p and miR-29a have been proved to function as predictive factors for assessing the 28-day mortality in patients with septic AKI [14]. Here, we tried to investigate the functions of miR-191-5p in sepsis-induced AKI. Remarkably, the biomarker roles of miR-191-5p have been demonstrated in many diseases, for instance, Kumar et al. [15] demonstrated that miR-191-5p functions as one of the best predictors for the Alzheimer’s disease with >95% accuracy. Similarly, in tuberculosis (TB), the expression of miR-191-5p showed an observable difference between patients with TB and healthy controls [16]. The previous study showed that compared with the healthy volunteers, miR-191-5p was remarkably down-regulated in patients with septic AKI. More importantly, there was also remarkable difference in miR-191-5p levels between septic AKI and sepsis-non AKI patients [17]. All of these data suggested the potential value of miR-191-5p in distinguishing septic AKI. Thus, the present study tried to investigate the role of miR-191-5p in the progression of septic AKI.

## Materials and methods

### Animal model construction

A total of 40 female Wistar rats (200–250 g) were obtained from Shanghai SLAC Laboratory Animal Co. Ltd. (Shanghai, China). The mimic control (Forward: 5′-UUCUCCGAACGUGUCACGUTT-3′ and Reverse: 5′-ACGUGACACGUUCGGAGAATT-3′) or miR-191-5p mimic (Forward: 5′-CAACGGAAUCCCAAAAGCAGCUG-3′, and Reverse: 5′-GCUGCUUUUGGGAUUCCGUUGUU-3′) were synthesized by GenePharma (Shanghai GenePharma Co., Ltd., Shanghai, China). Rats were equally assigned to four groups (*n*=10): Control group was a sham group; Cecal ligation and puncture (CLP) group were a model group; MC group were injected with mimic control based on CLP group, and M group were injected with miR-191-5p mimic based on CLP group. The rats of MC and M groups were injected with mimic control or miR-191-5p mimic from caudal vein before CLP operation. The CLP model was used to construct the sepsis animal model as previously described [18,19]. On the day of surgery, rats were anesthetized with 2.5% isoflurane (Sigma–Aldrich; Merck KGaA, Darmstadt, Germany). Following laparotomy, 1.5 cm of the cecal tip was ligated using 4-0 silk and punctured twice gently with an 18-gauge needle. Approximately 1 mm column of fecal material was squeezed from the punctures. In the control group, the cecum was isolated without ligation or puncture. After the operation, all the rats were fed conventionally.

### Sample preparation

Rats were anesthetized with 2.5% isoflurane (Sigma–Aldrich) and then killed at 12, 24 and 48 h after operation. After being perfused with phosphate buffered saline (PBS, pH 7.4), kidney tissues were harvested immediately. Part of kidney tissues was frozen in liquid nitrogen and stored at −80°C for subsequent immunoblotting, and some tissues were fixed in 4% (w/v) paraformaldehyde for histological examination. In addition, blood samples were collected by an aortic puncture.

### Measurement of white blood cell, serum creatinine and blood urea nitrogen

The white blood cells were counted using BC-2800 Fully Automatic Hematology Analyzer (Mindray). The blood was centrifuged (3000 rpm for 10 min at 4°C) and we measured the levels of serum creatinine (SCr) and blood urea nitrogen (BUN) as the important indexes of renal injury according to the protocols of kits (Institute of Jiancheng Bioengineering, Nanjing, China) with an AutoAnalyzer (Roche Diagnostics, Mannheim, Germany).

### Histological examination

For the observation of pathological changes, the collected kidneys were fixed in 4% (w/v) paraformaldehyde at 4°C for 24–48 h and embedded in paraffin and cut into 4-μm sections for Hematoxylin and Eosin (H&E) staining. The pathological changes of kidneys were examined and photographed using a light microscopy (Olympus America, Inc., NY, U.S.A.). The degree of kidney damage was estimated by the following criteria: 0, normal; 1, damage involving <25% of the tubules; 2, damage involving 25–50% of the tubules; 3, damage involving 50–75% of the tubules; and 4, damage involving 75–100% of the tubules.

### Enzyme-linked immunosorbent assay

The blood samples were centrifuged at 3000×***g*** for 15 min at 4°C for serum separation. The levels of serum tumor necrosis factor-α (TNF-α), interleukin (IL) 1β (IL-1β) and IL-6 were evaluated using different commercial Enzyme-linked immunosorbent assay (ELISA) kits (R&D Systems, Inc. MN, U.S.A.) following the manufacturer’s instructions. The absorbance of each well was determined using a microplate reader at 450 nm (Bio-Rad Laboratories, Inc., Hercules, CA, U.S.A.). The blank well was applied for zero calibration.

### Quantitative PCR

Total RNA was isolated from the collected kidney tissues by TRIzol (Invitrogen; Thermo Fisher Scientific, Rockford, IL, U.S.A.). Total RNA (1 μg) was introduced for reverse transcription with a PrimeScript RT Reagent Kit (Takara, Dalian, China). It is noted that, for the detection of mRNA quantification, the reverse transcription of cDNA was using oligo(dT)_18_ primer. Subsequently, the relative expression was determined by the SYBR Premix ExTaq (Takara) on the ABI 7500 Real-time PCR system (Applied Biosystems, Foster City, CA, U.S.A.). The amplification conditions were the following: 95°C for 1 min, 40 cycles of 95°C for 15 s, 55°C for 30 s and 72°C for 30 s. The expression of miR-191-5p was calculated according to the 2^−ΔΔ*C*^_t_ method [20]. The expressions of miRNA and cellular genes were normalized to those of U6 and GAPDH. All primers were listed in Table 1.

**Table 1:** Primers for RT-qPCR

Gene name	Primer sequences
miR-191-5p	Forward: 5′-CGGAATCCCAAAAGCAGCTG-3′
	Reverse: 5′-TGTCGTGGAGTCGGCAATTG-3′
Bcl-2	Forward: 5′-AGCCCTGTGCCACCTGTGGT-3′
	Reverse: 5′-ACTGGACATCTCTGCAAAGTCGCG-3′
Bax	Forward: 5′-AACAACATGGAGCTGCAGAGG-3′
	Reverse: 5′-GAAGTTGCCGTCTGCAAACAT-3′
Cleaved caspase-3	Forward: 5′-TGTGAGGCGGTTGTGGAAGAGT-3′
	Reverse: 5′-AATGGGGGAAGAGGCAGGTGCA-3′
OXSR1	Forward: 5′-AAAGACGTTTGTTGGCACCC-3′
	Reverse: 5′-GCCCCTGTGGCTAGTTCAAT-3′
GAPDH	Forward: 5′-TGATGACATCAAGAAGGTGG-3′
	Reverse: 5′-TTACTCCTTGGAGGCCATGT-3′
U6	Forward: 5′-ATGACGTCTGCCTTGGAGAAC-3′
	Reverse: 5′-TCAGTGTGCTACGGAGTTCAG-3′

Abbreviation: Bcl-2, B-cell lymphoma-2; OXSR1, oxidative stress responsive 1.

### Renal cell separation

A portion of collected kidney tissue from rats in MC and M groups was used for isolation of renal cells as previously described [21]. Briefly, the kidney tissues were cut into fragments and digested with collagenase IV (200 U/ml, Sigma–Aldrich) for 1 h under constant rotation at 37°C. Cells were centrifuged at 800×*g* at 4°C for 5 min and resuspended using RPMI-1640 medium (ATCC, Manassas, VA, U.S.A.) with 10% fetal bovine serum (ATCC). The cell suspension was passed through a 200-mesh sieve (75 μm) to remove the undigested tissue pieces. Renal cells were maintained in a 5% CO_2_ at 37°C for subsequent experiments.

### Luciferase reporter assays

With TargetScan prediction, the sequence covering the miR-191-5p target site in wild-type (WT) oxidative stress responsive 1 (OXSR1)-3′-untranslated region (UTR) was cloned into the pGL3-Basic (Promega, Madison, WI, U.S.A.). We also cloned the mutant OXSR1-3′-UTR (MUT) into pGL3 luciferase vector (Promega). Before cell transfection, renal cells collected from septic rats were seeded into 12-well plates (5 × 10^5^ cells/well) in CO_2_ at 37°C. Subsequently, pGL-3-OXSR1 WT or MUT vector (1 μg per well) was transfected into renal cells using Lipofectamine 2000 reagent (Invitrogen). Both Firefly and *Renilla* luciferase activities were determined 24 h after transfection by Dual-Luciferase Reporter Assay System kit (Promega).

### Cell transfection

In order to further confirm that the functions of miR-191-5p involved the repression of OXSR1, OXSR1 overexpression or negative control vector was transfected into rat-derived kidney cells. The full-length open reading frame of rat OXSR1 in pCMV6 was obtained from GenePharma, and the empty vector was set as negative control. Before transfection, renal cells isolated from rats of MC and M groups were seeded into 12-well plates (5 × 10^3^ cells/well). OXSR1 overexpression and negative control vector were synthesized by Shanghai GenePharma, Inc. (Shanghai, China). Cell transfection was carried out in accordance with Lipofectamine 2000 (Invitrogen). After 24 h of transfection, transfection efficiencies were monitored by Western blot to determine whether up-regulation was successful.

Renal cells from MC or M groups were divided into two subgroups for transfection. In MC + NC subgroup, renal cells isolated from MC group were transfected with NC vector. In MC + OXSR1 subgroup, renal cells of MC rat models were transfected with OXSR1 overexpression vector. In M + NC subgroup, renal cells isolated from M group were transfected with NC vector, and in M + OXSR1 subgroup, cells from M group were transfected with OXSR1 overexpression vector.

### Cell counting kit-8 assay

Cell counting kit-8 (CCK-8) assay (Beyotime Institute of Biotechnology, Haimen, China) was performed to measure cell viability of transfected cells. Approximately 5 × 10^3^ transfected cells were seeded into each well of a 96-well plate. Ten microliters of CCK-8 was added to each well every 24 h for 30-min incubation. Subsequently, the absorbance values of the experimental wells were analyzed by a microplate reader at 490 nm (Bio-Rad Laboratories, Inc.).

### Apoptosis rate analysis

The apoptosis rates were analyzed to reflect the effects of miR-191-5p/OXSR1 axis on renal cells of septic rat model. Briefly, renal cells were collected from every experimental group and washed with cold PBS, and then resuspended with 100 μl of 1× binding buffer. Next, 5 μl Annexin V-fluorescein isothiocyanate and 5 μl propidium iodide (Invitrogen) were added to each well. The plates were maintained in the dark at room temperature for 15 min. The plates were collected and analyzed by flow cytometer (Beckman Coulter, Inc., Brea, CA, U.S.A.).

### Western blot

Total protein was isolated from the transfected rat-derived renal cells using radioimmunoprecipitation assay buffer (Sigma–Aldrich). Equal amount of protein samples were separated by 10% sodium dodecyl sulfate/PAGE and transferred to polyvinylidene difluoride membranes (PVDF). For immunoblotting, 5% non-fat milk was used to block membranes at 4°C overnight, then membranes were incubated with the following primary antibodies, including rabbit anti-B-cell lymphoma-2 (Bcl-2, 1:1000, 26 kDa, #ab59348), rabbit anti-Bax (Rabbit, 1:2000, 21 kDa, #ab32503), rabbit anti-Cleaved Caspase-3 (C caspase-3, 1:1000, 17 kDa, #ab2302), rabbit anti-OXSR1 (1:1000, 58 kDa, #ab97694) and mouse anti-GAPDH (1:20000, 36 kDa, #ab8245). After being washed with PBS, membranes were cultured with a 1:20000 dilution of HRP–conjugated anti-rabbit (#ab205718, 42 kDa) or anti-mouse secondary antibody (#ab205719, 52 kDa) for 1 h at 4°C. The blots were detected by enhanced chemiluminescence (ECL, Millipore, U.S.A.). All antibodies used were obtained from Abcam (Cambridge, MA, U.S.A.).

### Statistical analysis

Data were expressed as mean ± SD. Differences between two groups were analyzed using Student’s *t* test, and one-way analysis of variance (ANOVA) followed by Dunnett’s *t* test was used to analyze statistical difference in mean values between multiple groups using SPSS version 19.0 (IBM SPSS, Armonk, NY, U.S.A.). *P*<0.05 was explained as statistically significant.

## Results

### Enhancing miR-191-5p diminished inflammatory response and cell apoptosis in the kidneys of septic rat models

**Figure 1:**
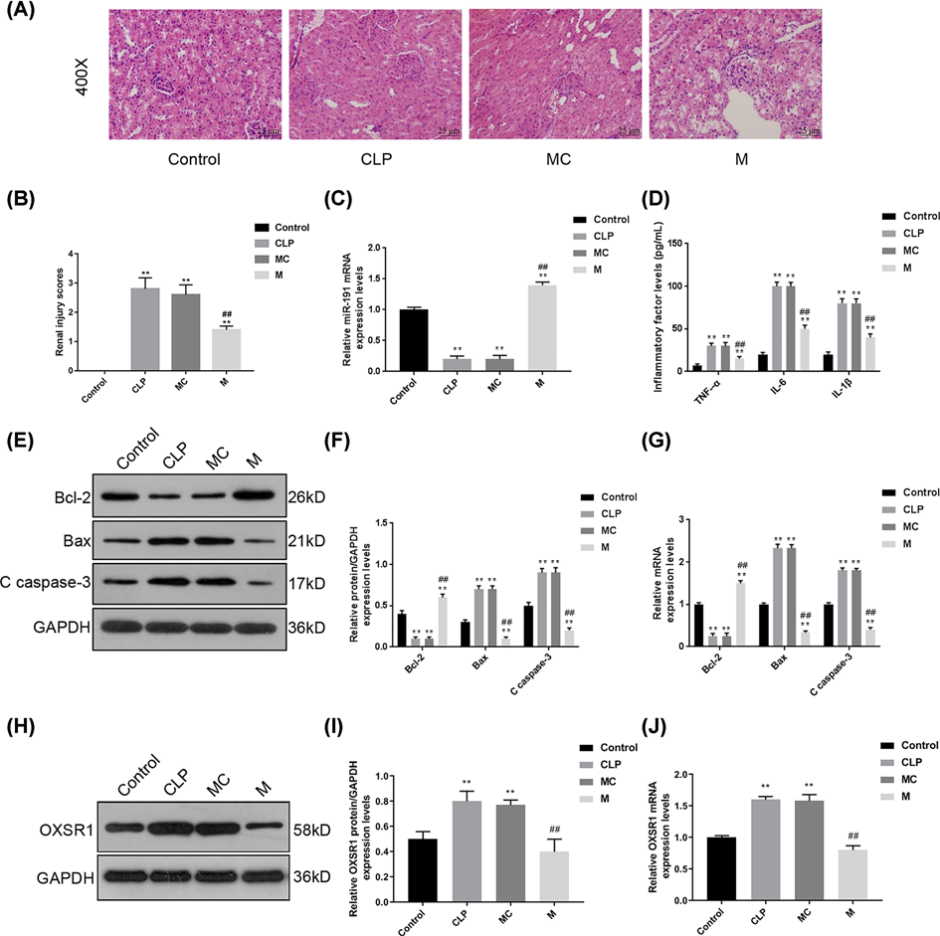
Enhancing miRNA-191-5p diminished inflammatory response and cell apoptosis in septic AKI rat models In order to confirm the functions of miR-191-5p in the progression of sepsis-induced AKI, we injected miR-191-5p mimic into rat models before CLP surgery. After 24 h of surgery, the blood samples and kidney tissues were collected for subsequent experiments. (**A**) After H&E staining, we observed the histopathological changes in the kidneys of septic AKI rat model with or without the injection of miR-191-5p. Original magnification: ×400. Calibration bar = 25 μm. (**B**) Semi-quantitative renal injury analysis was evaluated in H&E-stained renal tissue sections. (**C**) The levels of miR-191-5p in every experimental group were determined by quantitative PCR (qPCR). (**D**) The levels of serum TNF-α, IL-1β and IL-6 were analyzed by several commercial ELISA kits. (**E**–**J**) The mRNA and protein levels of Bcl-2, Bax, C caspase-3 and OXSR1 were measured by qPCR and Western blot, respectively. Each value represents mean ± SD (*n*=3). The expressions of cellular genes and miR-191-5p were normalized to GAPDH and U6, respectively. **P<0.01 vs. Control group; ^##^*P*<0.01 vs. MC group.

It is pivotal to authenticate whether sepsis and septic AKI were induced in CLP rats, so several biomarkers were detected at 12, 24 and 48 h after surgery. The white blood cell count, serum TNF-α and IL-6 level were elevated sharply in CLP rats at 24 h after surgery (Supplementary Figure S1A). Moreover, the serum BUN and SCr levels were also increased while the weights were reduced at 24 h after CLP (Supplementary Figure S1B). In order to confirm the functions of miR-191-5p in the progression sepsis-induced AKI, we observed the histopathological changes in the kidneys of septic rat models with or without the effects of miR-191-5p. According to the details in Figure 1A, compared with that in Control group, we found severe pathologic changes in CLP and MC groups. However, the pathologic changes were observably decreased in the M group. Meanwhile, the renal injury scores were much higher in CLP and MC groups, compared with that in the Control group, but injection of miR-191-5p significantly decreased the scores (*P*<0.01, Figure 1B). These data indicated that enhancing miR-191-5p levels could improve kidney injury *in vivo*. We further examined miR-191-5p levels, and serum inflammatory cytokines and apoptotic proteins in the kidneys of septic rats. First, the level of miR-191-5p was much reduced in the rats from CLP and MC group, and the injection of miR-191-5p mimic could significantly enhance the level of miR-191-5p (*P*<0.01, Figure 1C). As detailed in Figure 1D, the levels of serum TNF-α, IL-1β, and IL-6 were obviously increased in CLP and MC groups (*P*<0.01), the injection of miR-191-5p mimic had an inhibitory effect on these inflammatory cytokines (*P*<0.01). We additionally measured the protein levels of Bcl-2, Bax and C caspase-3 to assess the effects of miR-191-5p on cell apoptosis (Figure 1E–G). We found that the CLP surgery could induce the down-regulation of Bcl-2 and up-regulation of Bax and C caspase-3, but enhancing miR-191-3p levels could effectively inhibit the induction of surgery (*P*<0.01). Our results suggested that miR-191-5p overexpression could reduce renal injury scores, and improve sepsis-induced organ injury, inflammation and apoptosis. Then we detected the mRNA and protein levels of OXSR1, which were increased in the CLP rats and were repressed by miR-191-5p (Figure 1H–J).

### OXSR1 was identified as a target of miR-191-5p

**Figure 2:**
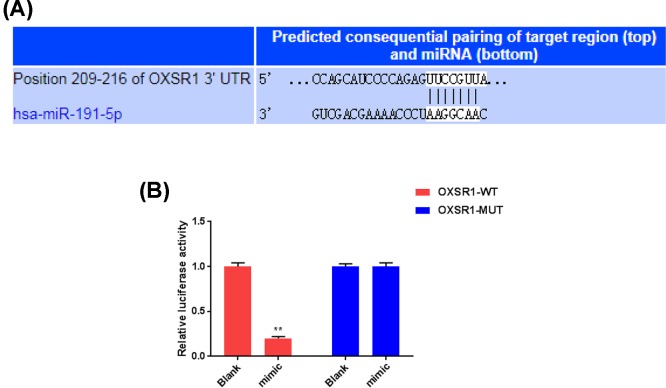
OXSR1 was identified as a target of miR-191-5p In order to investigate the mechanisms underlying the protective role of miR-191-5p in septic AKI rat models, we tried to identify the potential target of miR-191-5p. (**A**) With TargetScan prediction, fragment containing the specific binding site of miR-191-5p was found in OXSR1-3′-UTRs. (**B**) The correlation between OXSR1 and miR-191-5p was verified by dual-luciferase reporter system. Each value represents mean ± SD (*n*=3). **P<0.01 vs. Blank group.

We further investigated the mechanisms underlying miR-191-5p improving sepsis-induced kidney injury through screening the potential target of miR-191-5p. OXSR1 is revealed to play important roles in cytoskeleton rearrangements and control the proliferation or apoptosis of cells. Besides, the overexpression of OXSR1 was reported to be involved in less ischemia–reperfusion injury and delayed graft function. According to the prediction of TargetScan, the sequence of OXSR1-3′-UTR contains a 7-nucleotide binding site of miR-191-5p (Figure 2A). The luciferase reporter assay showed that miR-191-5p mimic remarkably decreased the luciferase activity of pGL3-OXSR1-WT, but failed to affect the luciferase activity of pGL3-OXSR1-MUT (Figure 2B). Thus, miR-191-5p could directly regulate the expression of OXSR1.

### The up-regulation of OXSR1 partially reversed the protective effects of miR-191-5p on rat-derived renal cells

**Figure 3:**
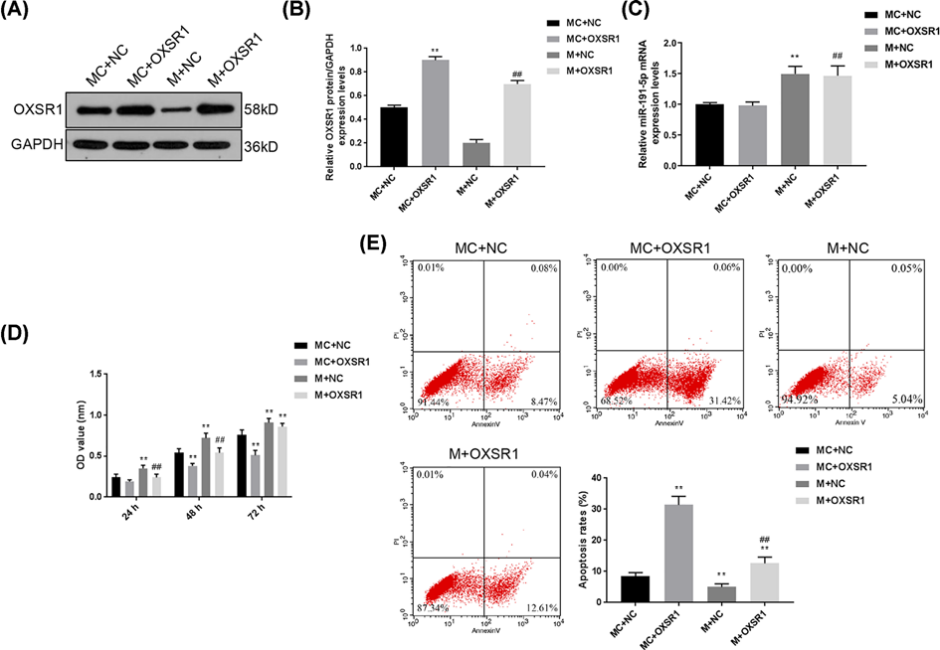
The up-regulation of OXSR1 partially reversed the protective effects of miR-191-5p on rat-derived renal cells OXSR1 overexpression and negative control vector were transfected into renal cells isolated from the rats of MC and M groups to further confirm the role of OXSR1 in the effects of miR-191-5p. (**A,B**) The protein levels of OXSR1 of different groups were determined by Western blot. (**C**) The expression of miR-191-5p of different groups were detected by qPCR. (**D**) The effects of OXSR1 overexpression on cell viability were assessed by CCK-8 assay. (**E**) The changes in apoptosis were analyzed by flow cytometry. Each value represents mean ± SD (*n*=3). The expressions of cellular genes were normalized to GAPDH. **P<0.01 vs. MC + NC group; ^##^*P*<0.01 vs. M + NC group.

The cells were isolated from the kidney tissues of septic rats. OXSR1 overexpression and negative control vectors were transfected into renal cells from septic rats. The results of transfection efficiencies of OXSR1 and NC vectors were presented in Figure 3A,B. The transfection of OXSR1 overexpression vector could obviously enhance the protein level of OXSR1 in MC + OXSR1 group. The OXSR1 protein level in M + NC group was much decreased compared with that in the MC + NC group, and OXSR1 transfection could significantly promote the expression of OXSR1 (*P*<0.01). Moreover, the miR-191-5p expression was not affected by the OXSR1 (Figure 3C). The CCK-8 assay showed that cell viabilities were positively related with the levels of miR-191-5p, and negatively related to the expression of OXSR1 (Figure 3D). Additionally, we also analyzed the changes in apoptosis rates (Figure 3E). Compared with that in MC + NC group, the apoptosis rate in MC + OXSR1 group had a sharp increase (*P*<0.01), and M +NC group had a much lower apoptosis rate. However, the transfection of OXSR1 overexpression vector could enhance the apoptosis rate again (*P*<0.01). These results implied that enhancing OXSR1 could significantly reverse the suppressive effects of miR-191-5p on cell viability and apoptosis.

### The functional effects of OXSR1 on renal cells were related with the activation of p38 MAPK/NF-κB signaling pathway

**Figure 4:**
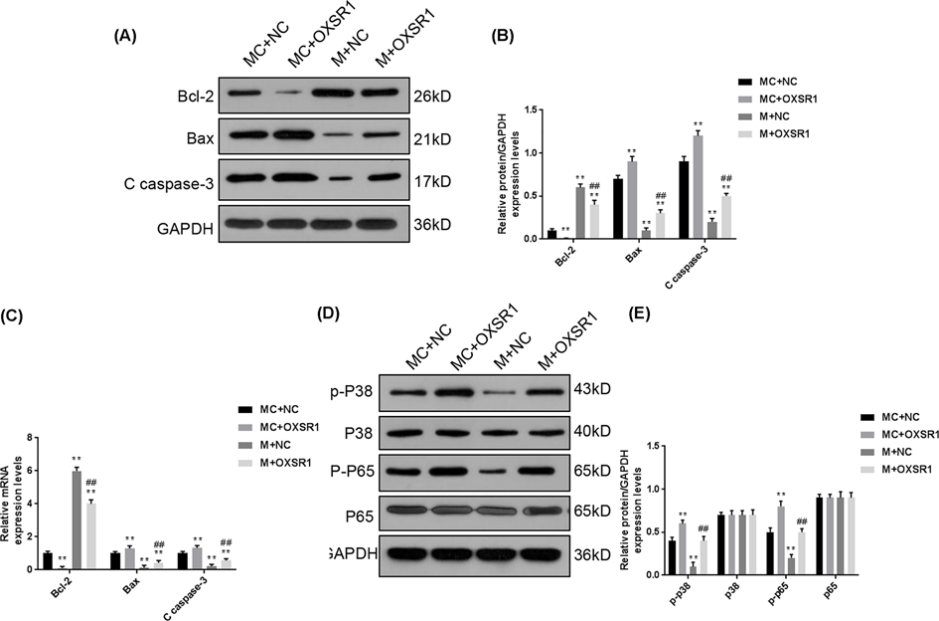
The functional effects of OXSR1 on renal cells were related with the activation of p38 MAPK/NF-κB signaling pathway We also further investigated the signaling pathway involved in the functions of OXSR1. (**A**–**C**) The protein and mRNA levels of several apoptotic genes were determined by Western blot and qPCR, including Bcl-2, Bax and C caspase-3. (**D,E**) The phosphorylation levels of p38 and p65 were measured to evaluate the activation of p38 MAPK/NF-κB signaling pathway. Each value represents mean ± SD (*n*=3). The expressions of cellular genes were normalized to GAPDH. **P<0.01 vs. MC + NC group; ^##^*P*<0.01 vs. M + NC group.

We also examined the protein and mRNA levels of apoptotic genes by quantitative PCR (qPCR) and Western blot (Figure 4A–C). In MC + OXSR1 group, the expression of Bcl-2 was much decreased, and the levels of Bax and C caspase-3 were much up-regulated, compared with the MC + NC group. The higher Bcl-2 level and lower Bax and C caspase-3 levels were found in M + NC group in comparison with those in MC + NC group (*P*<0.01). Consistent with the apoptosis, enhancing OXSR1 level could significantly inhibit the expression of Bcl-2 and enhancing Bax and C caspase-3 levels in M + OXSR1 group. Furthermore, we measured the phosphorylation levels of p38 and p65 to evaluate the activation of p38 MAPK/NF-κB signaling pathway (Figure 4D,E), and found that p-p38 and p-p65 were highly expressed in MC + OXSR1 group, lowly expressed in M + NC group. Importantly, the protein levels of p-p38 and p-p65 in M + OXSR1 group was much higher than that in M + NC group, indicating that enhancing OXSR1 expression could promote the activation of p38 MAPK/NF-κB signaling pathway.

## Discussion

Septic AKI is considered a major public health concern in today’s world, however, there is no effective treatment for it. Accumulation studies have demonstrated that the pathological process of septic AKI was highly associated with excessive inflammatory response and renal cell apoptosis [22,23]. Here, we investigated the functions of miR-191-5p in septic rat models and found that the injection of miR-191-5p mimic could observably inhibit renal injury scores. Meanwhile, the levels of inflammatory cytokines and apoptotic proteins both showed significant down-regulation under the effects of miR-191-5p mimic. These data suggested a protective role of miR-191-5p in the kidneys of septic rats. In order to further illustrate the mechanisms underlying the protective role of miR-191-5p in septic kidney injury, we focused on the target genes of miR-191-5p. With TargetScan prediction, miR-191-5p was confirmed to be able to specifically target OXSR1 mRNA. Renal cells isolated from the kidneys of septic rats in MC and M groups were used for cell transfection. After being transfected with OXSR1 overexpression vector, the cell viability and apoptosis were significantly increased. The up-regulated protein levels of p-p38 and p-p65 may suggest the involvement of p38 MAPK/NF-κB signaling pathway in the functions of OXSR1. Collectively, our results showed that the protective effects of miR-191-5p on kidney tissues of septic rats may rely on the repression of OXSR1, and enhancing OXSR1 level could abolish the functions of miR-191-5p mimic, and more aggravate cell apoptosis.

A great deal of evidence has suggested that the mechanisms underlying septic AKI involve the overactivation of the pro-inflammatory system [24]. The pro-inflammatory cytokines, such as TNF-α, IL-1β and IL-6, has been extensively proved to be involved in the pathogenesis of septic AKI [25]. Many previous studies have demonstrated that inhibiting the production of these pro-inflammatory cytokines could diminish the severity of septic kidney injury [26,27], for instance, miR-590-3p could effectively improve sepsis-induced kidney injury through negatively regulating the inflammatory response to septic challenge [28]. In our study, the injection of miR-191-5p mimic had a potently suppressive effect on the levels of serum TNF-α, IL-1β and IL-6 in septic rat models, which may indicate that miR-191-5p might be a possible therapeutic target for the treatment of septic AKI. Cell apoptosis is also a contributing factor that is strongly involved in the development of AKI, and many agents with the abilities of anti-inflammation and anti-apoptosis are promising for prevention of AKI [23,29]. Consistent with our results, the up-regulated Bcl-2 and down-regulated Bax and C caspase-3 in rat kidneys of M group may suggest that a portion of the protective effect of miR-191-5p on rat kidneys might be attributed to its ability to regulate apoptotic proteins.

Furthermore, it is known that almost all of the features of miRNAs are mediated through its ability to regulate downstream targets [30,31]. In the present study, OXSR1 was confirmed to be a potential target of miR-191-5p. OXSR1, a member of Ser/Thr kinase family, regulates downstream kinases in response to environmental stress, and it is responsible for ion co-transportation in the kidney [32,33]. The previous study showed that OXSR1 is responsible for the phosphorylation of the receptor expressed in lymphoid tissues (RELT) [34,35], and then the activation of RELT could promote cleavage of Caspase-3 and induce apoptosis of human epithelial cells in a novel extrinsic manner that is distinct from other death-inducing TNF receptor-associated factor (TNFR) members [36–38]. In our study, the obvious increased mRNA and protein levels of C caspase-3 after cells from M group were transfected with OXSR1 overexpression vector, which indicated that OXSR1 plays a promoting role in the progression of septic AKI. In addition, the previous study also showed that RELT could also recruit TNFR family members, and subsequently lead to the activation of the key factors of different signaling pathways [36,37], including p38 (p38 MAPK) and p65 (NF-κB), which both played important role in the progression of AKI [39,40]. Consistent with our data, the down-regulated phosphorylation levels of p38 and p65 in M group were observably up-regulated again with the increasing level of OXSR1. Taken together, based on the functions of OXSR1 in apoptosis and inflammation, the effects of miR-191-5p on the improvement of sepsis-induced kidney injury might be mainly attributed to inhibiting the expression of OXSR1.

## Conclusion

To conclude, this current study shows that the up-regulation of miR-191-5p can significantly suppress renal injury scores in septic rat models. Meanwhile, miR-191-5p is identified to be able to specifically target OXSR1. Considering the promoting effects of OXSR1 on inflammation and apoptosis, our results suggest that the protective effects of miR-191-5p on kidney cells were highly associated with the repression of OXSR1. These data provide a new perspective for understanding the mechanisms underlying septic AKI and suggest miR-191-5p could be a potential therapeutic target for the treatment of septic AKI.

## Supporting information

**Supplementary Figure S1:** 

## Abbreviations

AKI: acute kidney injury
ATCC: American Type Culture Collection
Bcl-2: B-cell lymphoma-2
BUN: blood urea nitrogen
C caspase-3: cleaved caspase-3
CCK-8: cell counting kit-8
CLP: cecal ligation and puncture
ELISA: enzyme-linked immunosorbent assay
GAPDH: glyceraldehyde-3-phosphate dehydrogenase
H&E: Hematoxylin and Eosin
HRP: horseradish peroxidase
IL: interleukin
miRNA: microRNA
OSR1: oxidative stress responsive 1
OXSR1: oxidative stress responsive 1
PBS: phosphate buffered saline
RELT: receptor expressed in lymphoid tissue
SCr: serum creatinine
TB: tuberculosis
TNFR: tumor necrosis factor receptor-associated factor
TNF-α: tumor necrosis factor-α
UTR: untranslated region
WT: wild-type
