# Supplementary figures and images for: MicroRNA-191-5p diminished sepsis-induced acute kidney injury through targeting oxidative stress responsive 1 in rat models
Source: Biosci Rep. 2019 Aug 13;39(8):BSR20190548. doi: 10.1042/BSR20190548 (PMC6692571; doi:10.1042/BSR20190548)

**A**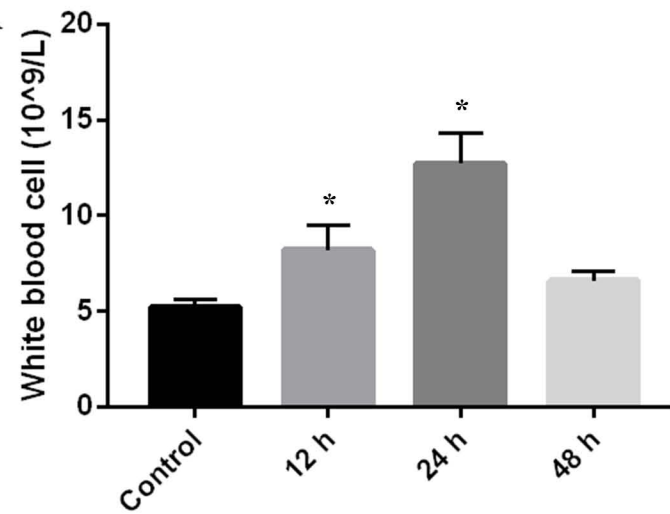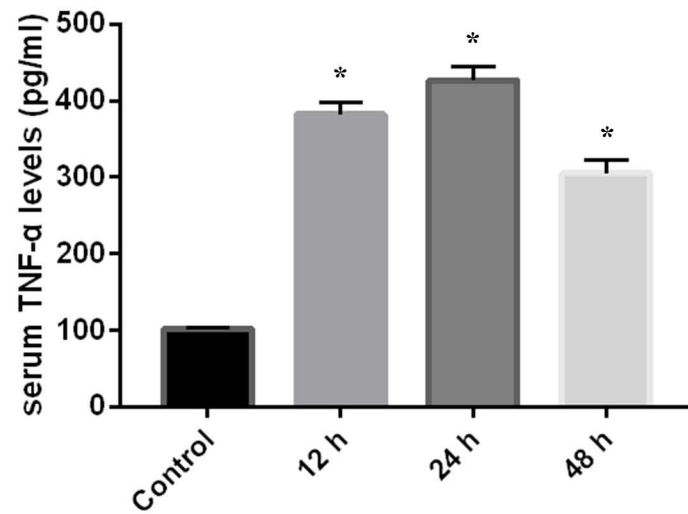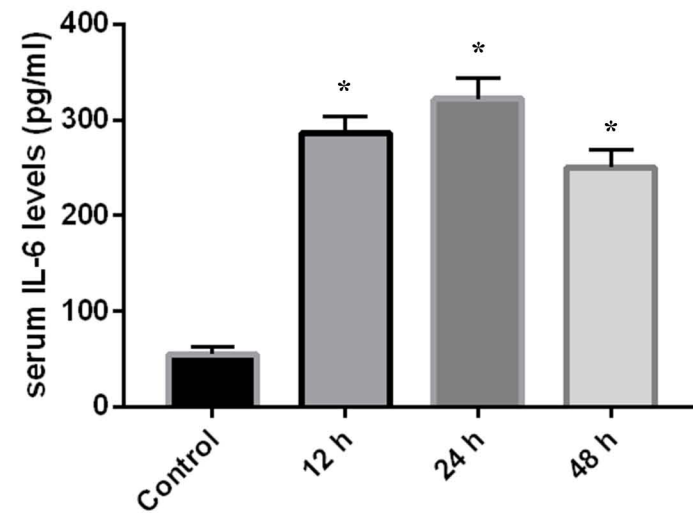**B**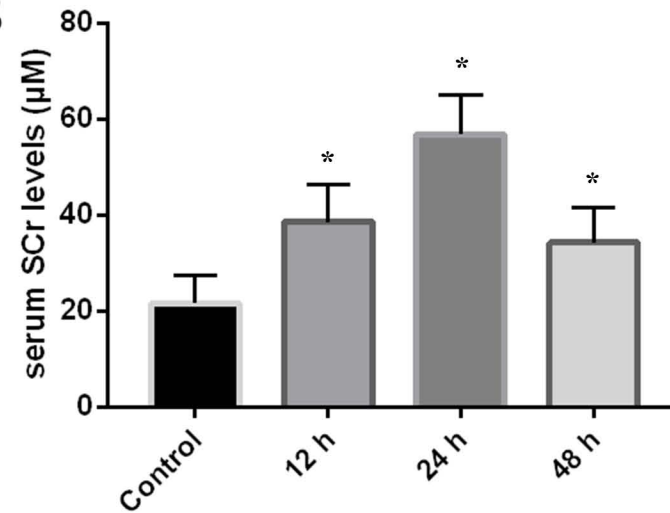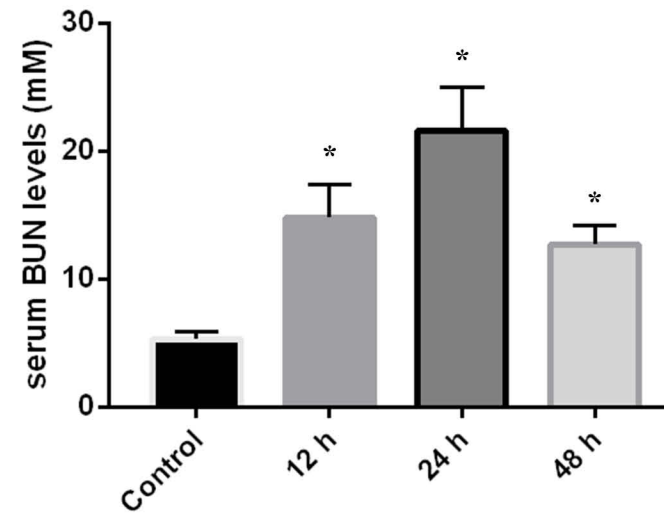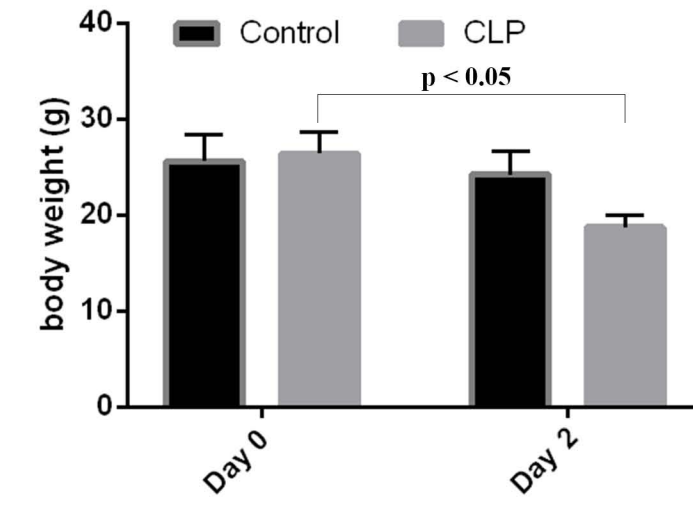

Supplement: Supplementary file 1 [file bsr20190548_Supp1.pdf]
